# Cryptic metabolisms in anoxic subseafloor sediment

**DOI:** 10.1111/1758-2229.12983

**Published:** 2021-06-28

**Authors:** Arkadiy I. Garber, Gustavo A. Ramírez, Sean M. McAllister, William Orsi, Steven D'Hondt

**Affiliations:** ^1^ School of Life Sciences, Arizona State University Tempe AZ USA; ^2^ Graduate School of Oceanography, University of Rhode Island Narragansett RI USA; ^3^ Department of Marine Sciences, University of North Carolina Chapel Hill NC USA; ^4^ College of Veterinary Medicine, Western University of Health Sciences Pomona CA USA; ^5^ Joint Institute for the Study of the Atmosphere and Ocean, University of Washington Seattle WA USA; ^6^ Pacific Marine Environmental Laboratory, National Oceanic and Atmospheric Administration Seattle WA USA; ^7^ Department of Earth & Environmental Sciences, Paleontology and Geobiology Ludwig‐Maximilians‐Universität München Munich 80333 Germany

## Abstract

Microbial gene expression in anoxic subseafloor sediment was recently explored in the Baltic Sea and the Peru Margin. Our analysis of these data reveals diverse transcripts encoding proteins associated with neutralization of reactive oxygen species, including catalase, which may provide an *in situ* source of oxygen. We also detect transcripts associated with oxidation of iron and sulfur, and with reduction of arsenate, selenate and nitrate. Given limited input of electron acceptors from outside the system, these results suggest that the microbial communities use an unexpectedly diverse variety of electron acceptors. Products of water radiolysis and their interactions with sediment continuously provide diverse electron acceptors and hydrogen. Cryptic microbial utilization of these oxidized substrates and H_2_ may be an important mechanism for multi‐million‐year survival under the extreme energy limitation in subseafloor sediment.

## Introduction

Marine sediment is estimated to host a third of Earth's Bacteria and Archaea (Kallmeyer *et al*., 2012; Bar‐On *et al*., 2018). Although cells are abundant, mean per‐cell rates of metabolic activity are generally very low in subseafloor sediment [>1 meter below seafloor (mbsf)] (D'Hondt *et al*., 2002; Jørgensen, 2011; Lomstein *et al*., 2012). Despite their low metabolic rates, cells inhabiting subseafloor sediment are generally alive (Schippers *et al*., 2005; Ramírez *et al*., 2018) and capable of growth (Morono *et al*., 2011).

In anoxic subseafloor sediment along continental margins, sulfate reduction, fermentation, and methanogenesis, fueled primarily by organic matter oxidation, are the dominant net metabolic activities (D'Hondt *et al*., 2002). Along continental margins, where rates of anaerobic activity are relatively high, detectable dissolved sulfate and net sulfate reduction are typically limited to sediment within and above the sulfate–methane transition zone (SMTZ) (D'Hondt *et al*., 2002; Orsi *et al*., 2013). Below this zone, dissolved sulfate is usually below detection and net respiration is primarily hydrogenotrophic methanogenesis (D'Hondt *et al*., 2002).

The energetic processes that enable long‐term microbial survival in deeply buried marine sediment are not fully understood. Long‐term survival in this habitat may be influenced by cryptic energy‐yielding activities that are not represented by net activities traditionally recognized from dissolved chemical profiles. For example, water radiolysis is a ubiquitous process in marine sediment (Sauvage *et al*., 2021). Water radiolysis, and the interaction of its products with sediment, generates diverse biologically accessible chemicals (Sauvage *et al*., 2021), leading us to hypothesize that radiolytic products may be used by sedimentary microbes for energy. To explore this possibility, we analysed the only publicly available metatranscriptomes from deep (5–159 mbsf range) anoxic sediment, sampled from the Peru Margin (Orsi *et al*., 2013) and the Baltic Sea (Zinke *et al*., 2017; Zinke *et al*., 2019). Using a custom set of hidden Markov models [HMMs (Johnson *et al*., 2010)], we targeted genes associated with reactive oxygen species neutralization and energy‐yielding reactions in deeply buried, energy‐starved, anoxic sedimentary environments.

## Results

### Geochemical profiles

**Fig 1:**
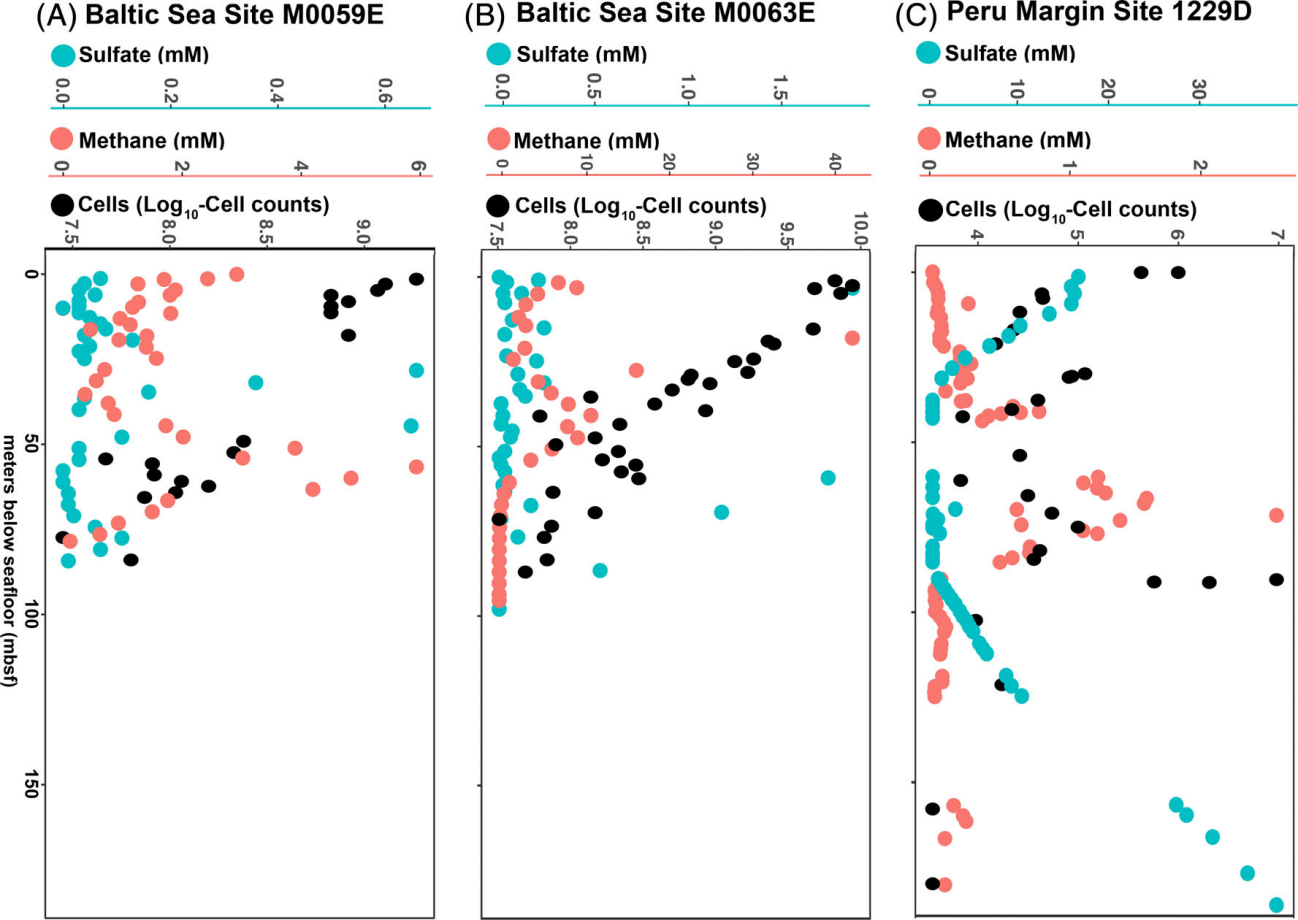
Sulfate (cyan), methane (red) and cell counts (black) profiles for sites (A) M0059E, (B) M0063E and (C) 1229D. Data from Expedition 347 Science Party (2015a), Expedition 347 Science Party (2015b) and Shipboard Scientific Party (2003).

Geochemical profiles show that all sites in this study are anoxic (Fig. 1). Detailed geochemical descriptions are found elsewhere (Shipboard Scientific Party, 2003;Expedition 347 Science Party, 2015a; Expedition 347 Science Party, 2015b). Briefly, at Baltic Sea Integrated Ocean Drilling Program Sites M0059 and M0063 (Fig. 1A and B), sulfate disappearance in the shallowest sediment (Expedition 347 Science Party, 2015a; Expedition 347 Science Party, 2015b) suggests that dissolved inorganic carbon (DIC) is the predominant net electron acceptor throughout most of the sediment column. At Peru Margin Ocean Drilling Program Site 1229 (Fig. 1C), sulfate penetrates deeper, and, at greater depths, diffuses upward from brine in deeper sediment (Shipboard Scientific Party, 2003). Sulfate is below detection and methane concentrations are high between 30 and 90 mbsf at Site 1229, indicating net methanogenesis (net DIC reduction) between those depths.

### Energy‐yielding reactions

**Fig 2:**
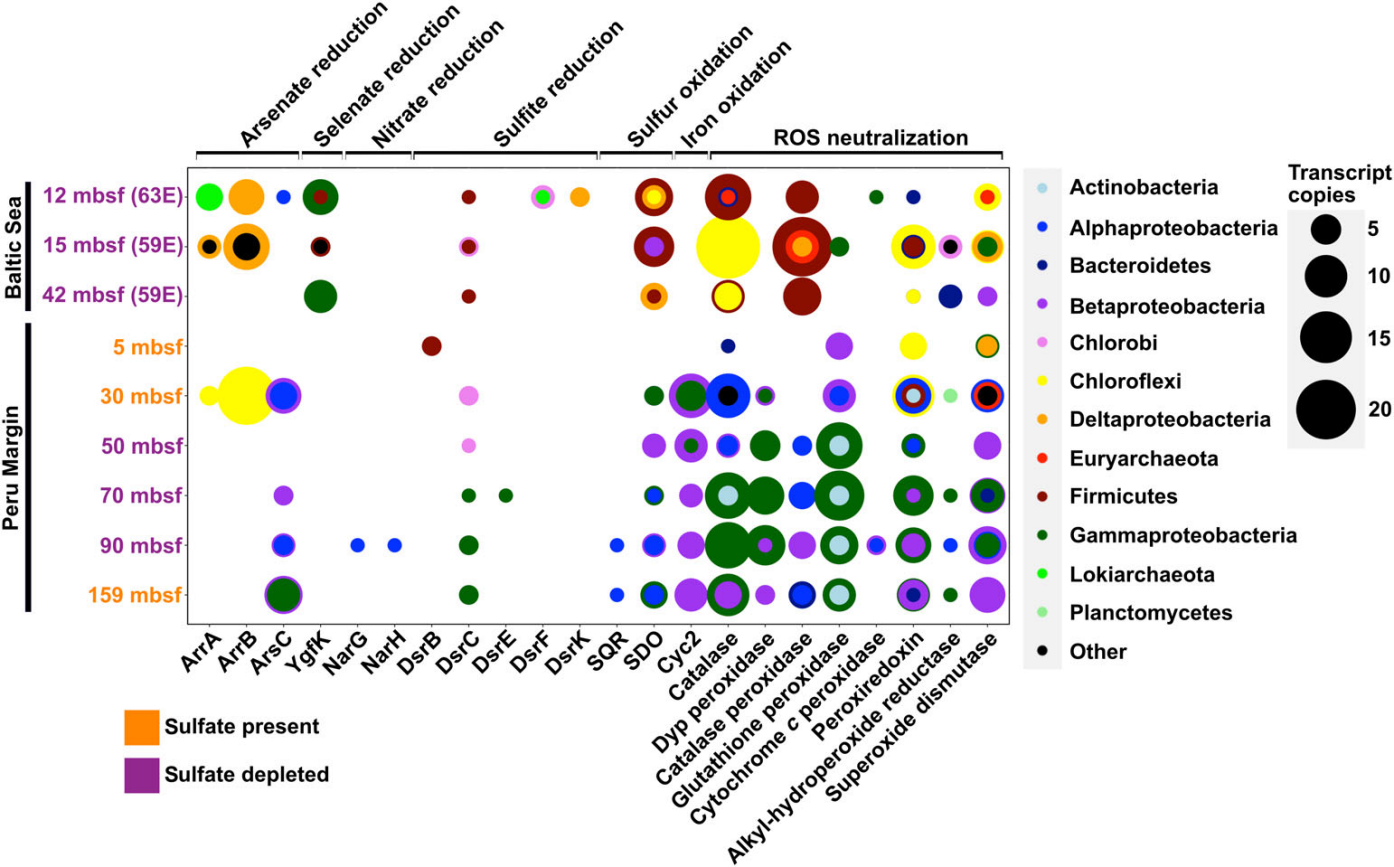
Dot plots showing lineage‐specific expression levels of PETs related to catabolic metabolisms and ROS neutralization. Transcripts from different taxonomic groups are colour‐coded at the phylum level (Class level for Proteobacteria). Sites are depicted by depth in meters below the seafloor (mbsf) and colour coded by sulfate presence (orange) or depletion (purple). The dot sizes represent transcript copies, which represent the number of gene variants of each gene detected in the metatranscriptomes.

We detected protein‐encoding transcripts (PETs) linked to diverse energy‐yielding metabolisms, derived largely from Chloroflexi and Firmicutes in the Baltic Sea subseafloor sediment and Actinobacteria and Proteobacteria in the Peru Margin subseafloor sediment (Fig. 2). Among these are PETs related to the reduction of sulfite, nitrate (as reported by Orsi *et al*., 2013), arsenate and selenate. In the Peru Margin sediment, iron oxidation is predicted at depth from the presence of the *cyc2* gene fragments (McAllister *et al*., 2020). Sulfur oxidation is predicted from the presence of *sdo* [sulfur dioxygenase (Liu *et al*., 2014)] and *sqr* [sulfide‐quinone oxidoreductase (Griesbeck *et al*., 2002)] gene transcripts in the Baltic Sea sediment (12–42 mbsf) and in all but one sample (5 mbsf) from the Peru Margin. Our detection of *sdo* transcripts is consistent with recent radiotracer‐based recognition of a cryptic sulfur cycle in ‘sulfate‐depleted’ marine sediment (Jørgensen *et al*., 2019).

### Reactive oxygen species neutralization

We detected PETs for neutralization of ROS in all surveyed metatranscriptomes (Fig. 2). PETs were identified for diverse ROS neutralization genes, including peroxiredoxin, hydroperoxide reductase and superoxide dismutase (Fig. 2). PETs homologous to the dye‐decolorizing peroxidase (Dyp), which performs hydrogen peroxide‐dependent oxidation of recalcitrant carbon polymers such as lignin (Chen *et al*., 2015), and heme peroxidase (Hansel *et al*., 2012), implicated in indirect extracellular manganese oxidation by *Roseobacter* spp. (Andeer *et al*., 2015), were also detected. Predicted taxonomic affiliations of transcripts indicate lineage‐specific expression of ROS‐related PETs (Fig. 2). Firmicutes, Chloroflexi and Proteobacteria drive expression patterns. Cyanobacteria, Chlorobi, Bacteroides, Aquificae and various Archaeal PETs are present but rare. Baltic Sea ROS‐related PETs belong to Firmicutes, Chloroflexi, and, to a smaller degree, Proteobacteria. In the Peru Margin, catalase transcripts are derived mostly from Proteobacteria.

## Discussion

### Catabolic activities

PETs related to the oxidation of iron and sulfur were detected in our survey (Fig. 2). In principle, sulfur can be oxidized with manganese or iron oxides as the electron acceptor (Jørgensen *et al*., 2019). In contrast, energy‐yielding microbial oxidation of iron typically requires highly oxidized electron acceptors, such as oxygen, hydrogen peroxide, superoxide and nitrate (Jørgensen *et al*., 2020). However, all of these samples are from much greater sediment depths than oxygen and nitrate appear to penetrate from the overlying ocean (D'Hondt *et al*., 2004). And while oxidized manganese can also serve as an electron acceptor for oxidizing iron, there is no evidence, as far as we know, that Mn(IV) and Fe(II) can be metabolically coupled by microorganisms. We also found *nar* transcripts exclusively in the 91‐mbsf Peru Margin sample, the approximate depth where downward‐diffusing methane is intercepted by sulfate diffusing up from brine below (D'Hondt *et al*., 2004), corroborating a previous report (Orsi *et al*., 2013). Overall, our survey of catabolic metabolisms suggests the presence of a cryptic source of oxidizing power in deep anoxic sediment.

### ROS‐related activities

The presence of ROS‐related PETs in deep sediment also indicates widespread neutralization and use of ROS in this system. The detection of transcripts encoding dye‐decolorizing peroxidase suggests the presence and microbial use of H_2_O_2_ to oxidize substrates and degrade recalcitrant organic polymers (Chen *et al*., 2015). Moreover, heme peroxidases, thought to be secreted from cells, react with H_2_O_2_ and produce superoxide, which can then oxidize Mn(II) to Mn(IV). Interestingly, fungal peroxidases, including manganese and heme peroxidases, are also thought to result in extracellular manganese oxidation (Hansel *et al*., 2012). Our observations, despite the known limitations of transcriptional surveys (see Notes on transcriptomic interpretation in the SI), support that H_2_O_2_ and perhaps other, more transient, ROS are present, at least ephemerally, in this ecosystem.

### Potential source of oxidants

We report the presence of PETs associated with neutralization of ROS, as well as PETs associated with reduction of arsenate, selenate and nitrate in deep subseafloor sediment, where DIC and sulfate are typically assumed to be the primary terminal electron acceptors. PETs associated with oxidation of sulfur and iron are also expressed, indicating that these communities are primed to utilize these energy‐generating pathways. We have considered whether these results could reflect signatures left over from the oxygenated world (i.e. constitutive functional gene expression rather than a true physiological response to the environment). Constitutive expression of functional genes from early in the sediment history might conceivably occur if microbes persist from that time without reproduction (D'Hondt *et al*., 2014; Kirkpatrick *et al*., 2019). However, recent studies have identified active cell division by dominant lineages of anaerobic subseafloor microbes (Atribacteria and Chloroflexi) in subseafloor sediment as old as 8 million years (Vuillemin *et al*., 2020a; Vuillemin *et al*., 2020b). In actively reproducing cells, the transcriptomic profiles change as net growth increases (Vuillemin *et al*., 2020a, Vuillemin *et al*., 2020b) and constitutive expression of functional genes should be a minor component of the transcriptome compared to the transcriptome that is activated as a part of the true physiological response to the environment. In accordance with this line of reasoning, residual transcription of oxygen utilization genes (e.g. terminal oxidases) is not observed in our analysis, indicating that transcripts not useful in the anoxic sediment are either not expressed or expressed at levels below detection limit.

Our observations collectively suggest the presence of an *in situ* source of biologically available oxidizing power in deep anoxic sediment. Although redox‐active antimicrobials (RAAs) might explain PETs for ROS (Imlay, 2008), the independent detection of PETs related to diverse catabolic redox activities suggests that the ROS transcriptional patterns may not be exclusively driven by RAAs. Unexpected redox processes can be directly and indirectly supported by water radiolysis, which is ubiquitous in marine sediment and other wet geological environments (Lin *et al*., 2006; Blair *et al*., 2007). Water radiolysis appears to be the predominant source of electron donors (H_2_) and diverse electron acceptors in marine sediment older than a few million years (Sauvage *et al*., 2021). Reaction of oxidized radiolytic products with sedimentary minerals, as well as various catalases and peroxidases, may generate redox species that are otherwise generally absent from sediment beneath the SMTZ [e.g. hydrogen peroxide, sulfite/sulfate, iron(III)].

### Concluding remarks

Our results provide evidence for potential cryptic redox cycling in deeply buried marine sediment. Using a small, highly curated database of marker genes of biogeochemical significance, we predict the occurrence of unexpectedly diverse microbial activities, including various forms of catabolic metabolism and oxidative defence, that go undetected by standard geochemical measurements in this habitat. This study greatly expands the potential metabolic diversity associated with anoxic subseafloor sediment and highlights reactions that may provide long‐term (multi‐million‐year scale) support for microbial communities buried in deep anoxic sediment.

## Materials and methods

### Sample collection

Information regarding collection, handling and processing of samples for the original transcriptomic studies can be found in the following publications: Peru Margin ‐ Orsi *et al*., 2013; Baltic Sea ‐ Zinke *et al*., 2017; Zinke *et al*., 2019 (see the Extended Methods section of the SI for additional details).

### Data acquisition and processing

Metatranscriptomic data from Peru Margin and Baltic Sea marine sediment were downloaded from the Sequence Read Archive (SRA) using the *SRA Toolkit* v.2.8.2 (SRA Toolkit Development Team, NCBI). Site metadata, sequence trimming, assembly and read map details are provided in the Extended Methods section of the SI.

### Annotation

To identify transcripts relevant to cryptic metabolisms and ROS neutralization, we developed two novel bioinformatics tools: *LithoGenie* and *RosGenie*. These software packages and the HMM libraries employed by each tool are public: https://github.com/Arkadiy-Garber/LithoGenie and https://github.com/Arkadiy-Garber/RosGenie. Software details, including marker gene selection, HMM calibrations and contaminant screening are provided in the Annotation section of the SI.

## Funding Information

G.A.R. was funded by an NSF C‐DEBI post‐doctoral fellowship. S.D. and G.A.R. were funded by the U.S. National Science Foundation through grants NSF‐OCE‐1130735 and NSF‐OCE‐0939564. S.M.M. was funded by the Joint Institute for the Study of the Atmosphere and Ocean (JISAO) under the NOAA Cooperative Agreement NA15OAR4320063.

## Author Contributions

G.A.R., S.D. and A.I.G. conceived the study; A.I.G. and G.A.R. performed bioinformatics analyses; A.I.G., G.A.R., S.M.M., W.O. and S.D. interpreted data and wrote the manuscript.

## Supporting information


**Appendix S1**. Supporting information.
