# Supplementary material for: Cryptic metabolisms in anoxic subseafloor sediment
Source: Environ Microbiol Rep. 2021 Jun 28;13(5):696–701. doi: 10.1111/1758-2229.12983 (PMC8518782; doi:10.1111/1758-2229.12983)
Supplement: Supplementary file 1 — Appendix S1. Supporting information. [file EMI4-13-696-s001.docx]

*Supplemental Information*

**Cryptic metabolisms in anoxic subseafloor sediment**

Arkadiy I. Garber^1*^, Gustavo A. Ramírez^2,3,4*†^, Sean M. McAllister^5,6^, William Orsi^7^, and Steven D’Hondt^2^

1: Arizona State University, School of Life Sciences, Tempe, AZ, USA.

2: University of Rhode Island, Graduate School of Oceanography, Narragansett, RI, USA.

3: University of North Carolina, Department of Marine Sciences, Chapel Hill, NC, USA.

4: Western University of Health Sciences, College of Veterinary Medicine, Pomona, CA, USA.

5: University of Washington, Joint Institute for the Study of the Atmosphere and Ocean, Seattle, WA, USA.

6: National Oceanic and Atmospheric Administration, Pacific Marine Environmental Laboratory, Seattle, WA, USA.

7: Department of Earth & Environmental Sciences, Paleontology and Geobiology, Ludwig-Maximilians-Universität München, 80333 Munich, Germany.

*: Both authors contributed equally and are considered co-first authors.

†: Corresponding author

ORCIDs:

GAR: 0000-0001-8122-4898

AIG: 0000-0001-7935-0246

SD: 0000-0001-9915-1148

WO: 0000-0002-4094-5637

**Extended Methods:**

*Core descriptions, sequence quality control, and gene feature generation*

Samples were collected during ODP Expedition 201 (Peru Margin: [1]), IODP Expedition 329 (Baltic Sea:[2, 3]). Samples were frozen at -80˚C on ship immediately after core retrieval and shipped to land-based laboratories on dry ice [4-6]. **Supplemental Table 1** shows the metadata (including sample depths, site names, and accession numbers) of datasets used in this study. The FASTQ reads were quality-trimmed using *Trimmomatic* v.0.36 (ILLUMINACLIP:TruSeq3-PE:2:30:10 LEADING:3 TRAILING:3 SLIDINGWINDOW:4:15 MINLEN:36) [7], and the surviving paired reads were combined using *Flash* v.1.2.11 (default settings) [8]; the extended and uncombined transcript fragments were then assembled using the *Trinity* v.2.1.1 assembler (default settings) [9]. Open reading frames (ORFs) from the assembled transcripts were predicted and translated to amino acid sequences using *Prodigal* v.2.6.3 with the ‘-p meta’ option [10].

*Annotation*

*MagicLamp* uses *HMMER* v.3.1b2 (*hmmsearch*) [11], and custom libraries of HMMs associated with genes of interest. This custom HMM library consists of models taken from TIGRFAMS and Pfam databases. HMMs were also taken from <https://github.com/kanantharaman/metabolic-hmms> [12] and others were constructed and calibrated specifically for this study. To construct HMMs, marker genes of interests were identified, and homologs extracted from RefSeq and UniProtKB. From manually-curated multiple sequence alignments, created using *Muscle* v.3.8.1551 [13] and visualized in AliView [14], HMMs were constructed using *HMMER* v.3.1b2 (*hmmbuild*) [11]. HMMs were calibrated by querying each HMM (*hmmsearch*) against NCBI’s RefSeq database. Results were then visually inspected to determine the sensitivity of the HMM and the optimum bit score cut off to be used when querying the model against the metatranscriptome assemblies.

*MagicLamp’s* “LithoGenie” library was used to identify PETs related to cryptic energy-generating reactions. *MagicLamp’s* “RosGenie” library was used to identify PETs related to reactive oxygen species neutralization. We carefully screened the annotated PETs from sedimentary datasets for potential contaminants. To identify transcripts potentially sourced from laboratory and/or reagent contaminants, we queried, using DIAMOND [15], all transcripts against a custom database of genes from common laboratory and reagent contaminants [16]. This database is based on a comprehensive analysis, carried out by Salter et al. [17], of contaminants in molecular kits and reagents, and includes genes from bacterial phyla, such as *Acinetobacter* and *Streptococcus*. Additionally, this database includes genes from microbial eukaryotes, such as diatoms. All ORFs with a known and common laboratory/reagent contaminant as a top hit (minimum amino acid similarity = 30%, minimum alignment length = 30 amino acids) are conservatively considered to be derived from contaminating organisms and were, thus, removed from further analysis. The remaining highly curated PETs can be interpreted as true positives that derive from active microbes within the deep anoxic sediment. The accuracy of this taxonomy-based de-contamination approach was tested with two genomes of isolate organisms and found to be >90% accurate in identifying the correct taxonomic affiliation (**Supplemental Figure 1**).

*Notes on transcriptomic interpretations*

We cannot definitively conclude that PETs associated with the cryptic metabolisms identified here are translated into proteins that are subsequently used for their predicted purposes. Nevertheless, detection of transcripts encoding marker genes for this broad range of cryptic activities (e.g., ROS neutralization, nitrate reduction, oxidation of iron and sulfur), strongly suggests that this community is primed to carry out these metabolic activities. We hypothesize that in an extremely energy-limited environment, it is unlikely that cells would expend energy to express diverse metabolic genes that are not translated into proteins. Moreover, the expression levels of transcripts related to ROS neutralization, and the redox cycling of sulfur, iron, and nitrogen are above the mean and median expression levels of genes in these metatranscriptomes (**Table 1**), suggesting that these metabolic processes may be significant. It is possible that these activities are sustained by continuous *in situ* creation of radiolytic products [18, 19]. In this manner, they may facilitate microbial community survival for millions of years following sediment deposition and burial.

| Location | Depth (mbsf) | Core | Number of Reads (HQ) | Number of predicted ORFs | SRA Accession | Article |
| --- | --- | --- | --- | --- | --- | --- |
| Peru Margin | 5 | 1229D | 79,919,957 | 43,955 | SRA058813 | Orsi *et al*., 2013 |
| Peru Margin | 30 | 1229D | 69,003,878 | 302,925 | SRA058813 | Orsi *et al*., 2013 |
| Peru Margin | 50 | 1229D | 65,572,971 | 59,405 | SRA058813 | Orsi *et al*., 2013 |
| Peru Margin | 70 | 1229D | 40,149,623 | 65,898 | SRA058813 | Orsi *et al*., 2013 |
| Peru Margin | 90 | 1229D | 49,925,920 | 66,259 | SRA058813 | Orsi *et al*., 2013 |
| Peru Margin | 159 | 1229D | 40,924,403 | 75,091 | SRA058813 | Orsi *et al*., 2013 |
| Baltic Sea | 12 | 63E | 16,497,274 | 184,859 | None found | Zinke *et al*., 2017 |
| Baltic Sea | 15 | 59E | 15,751,082 | 315,661 | None found | Zinke *et al*., 2017 |
| Baltic Sea | 42 | 59E | 14,399,563 | 80195 | None found | Zinke *et al*., 2017 |

**Table S1**: Summary of metatranscriptomic datasets used in this study, including the number of adaptor and quality-trimmed reads used, total number of predicted open reading frames (ORFs) generated from the assemblies, NCBI Short Read Archive (SRA) accession numbers, and the original publication for each dataset.

| **Site** | **Meters below seafloor** | **Median expression (Transcripts per million)** | **Mean expression (Transcripts per million)** |
| --- | --- | --- | --- |
| Baltic Sea (site 59E) | 15 | 1.1 | 5.2 |
| Baltic Sea (site 59E) | 42 | 3.0 | 22.5 |
| Baltic Sea (site 63E) | 12 | 1.8 | 10.6 |
| Peru Margin (site 1229D) | 5 | 21.2 | 24.8 |
| Peru Margin (site 1229D) | 30 | 2.9 | 3.5 |
| Peru Margin (site 1229D) | 50 | 14.4 | 18.1 |
| Peru Margin (site 1229D) | 70 | 10.7 | 16.2 |
| Peru Margin (site 1229D) | 90 | 10.8 | 15.9 |
| Peru Margin (site 1229D) | 159 | 9.9 | 14.0 |

**Table S2**: Mean and median expression levels of all transcripts combined from each metatranscriptomic dataset.


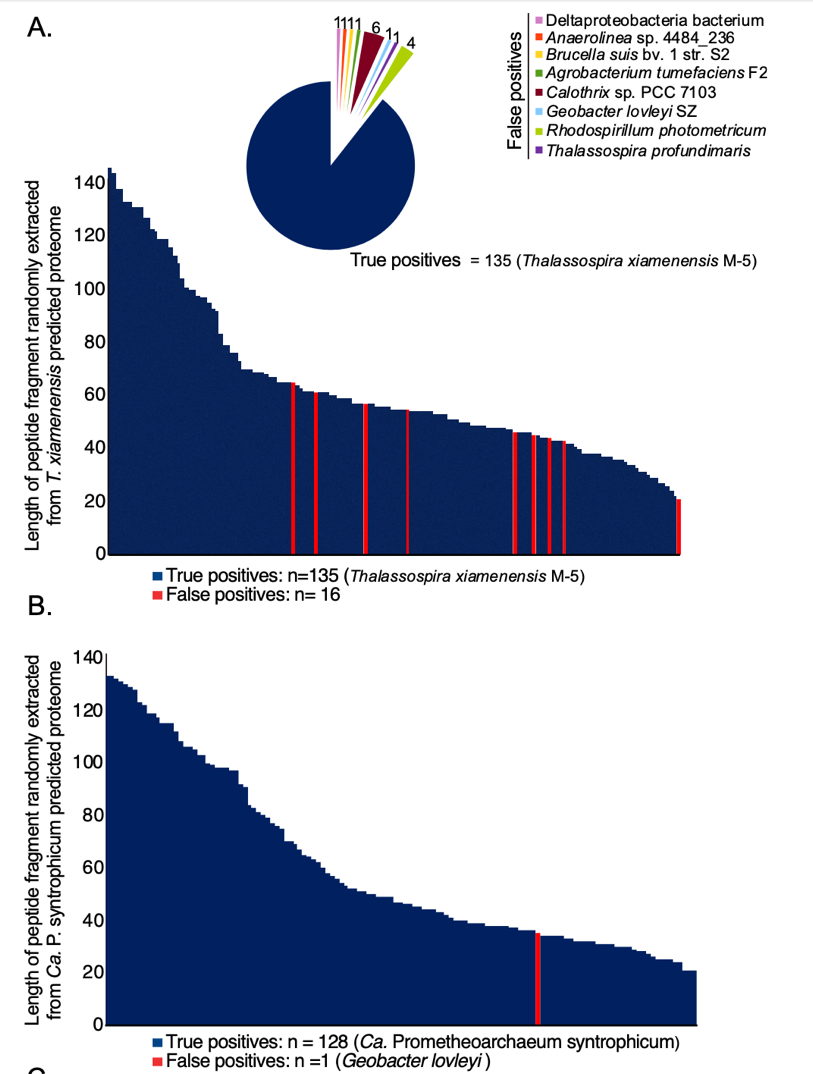


**Supplemental Figure 1** | An *in silico* experiment testing the rate of true positives and false negative annotations of 150 simulated ORFs (randomly selected peptide fragments) from the predicted proteomes of a bacterium (*T. xiamenensis*) and archaeon (*Ca*. Prometheoarchaeum syntrophicum). Panel A demonstrates that 89% of simulated ORFs (n=150 randomized peptide fragments) are correctly annotated to the species level. The same experiment applied to the predicted proteome of archaeon Ca. Prometheoarchaeum syntrophicum (Panel B) shows that the false negative rate is much lower: only one out of 150 peptide fragments (0.7%) was incorrectly assigned.

**Works cited**

1. ShipboardScientificPartySite1229. Site 1229. In D'Hondt, SL, Jørgensen, BB, Miller, DJ, et al, Proc ODP, Init Repts, 201: College Station, TX (Ocean Drilling Program), 1–78 2003. doi: doi:10.2973/odp.proc.ir.201.110.2003.

2. ShipboardScientificPartySiteM0059. Site M0059. In Andrén, T, Jørgensen, BB, Cotterill, C, Green, S, and the Expedition 347 Scientists, Proc IODP, 347: College Station, TX (Integrated Ocean Drilling Program). 2015. doi: doi:10.2204/​iodp.proc.347.103.2015

.

3. ShipboardScientificPartySiteM0063. Site M0063. In Andrén, T, Jørgensen, BB, Cotterill, C, Green, S, and the Expedition 347 Scientists, Proc IODP, 347: College Station, TX (Integrated Ocean Drilling Program). 2015. doi: doi:10.2204/​iodp.proc.347.107.2015

.

4. Zinke LA, Mullis MM, Bird JT, Marshall IPG, Jørgensen BB, Lloyd KG, et al. Thriving or surviving? Evaluating active microbial guilds in Baltic Sea sediment. Environ Microbiol Rep. 2017;9(5):528-36. doi: 10.1111/1758-2229.12578. PubMed PMID: 28836742.

5. ShipboardScientificPartyExplanatoryNotes. Leg 201. In D'Hondt, SL, Jørgensen, BB, Miller, DJ, et al, Proc ODP, Init Repts, 201: College Station, TX (Ocean Drilling Program) 1-103. 2003.

6. D’Hondt S, Inagaki F, Zarikian CA, Abrams LJ, Dubois N, Engelhardt T, et al. Presence of oxygen and aerobic communities from sea floor to basement in deep-sea sediments. Nature Geoscience. 2015;8(4):299-304. doi: 10.1038/ngeo2387.

7. Bolger AM, Lohse M, Usadel B. Trimmomatic: a flexible trimmer for Illumina sequence data. Bioinformatics. 2014;30(15):2114-20. doi: 10.1093/bioinformatics/btu170. PubMed PMID: 24695404; PubMed Central PMCID: PMCPMC4103590.

8. Magoc T, Salzberg SL. FLASH: fast length adjustment of short reads to improve genome assemblies. Bioinformatics. 2011;27(21):2957-63. doi: 10.1093/bioinformatics/btr507. PubMed PMID: 21903629; PubMed Central PMCID: PMCPMC3198573.

9. Haas BJ, Papanicolaou A, Yassour M, Grabherr M, Blood PD, Bowden J, et al. De novo transcript sequence reconstruction from RNA-seq using the Trinity platform for reference generation and analysis. Nat Protoc. 2013;8(8):1494-512. doi: 10.1038/nprot.2013.084. PubMed PMID: 23845962; PubMed Central PMCID: PMCPMC3875132.

10. Hyatt D, Gwo-Liang C, LoCascio P, Land M, Larimer F, Hauser L. Prodigal- prokaryotic gene recognition and translation initiation site identification. BMC Bioinformatics. 2010;11(119).

11. Johnson L, Eddy S, Portugaly E. Hidden Markov model speed heuristic and iterative HMM search procedure. BMC Bioinformatics. 2010;11(431). doi: <http://www.biomedcentral.com/1471-2105/11/431>.

12. Anantharaman K, Brown CT, Hug LA, Sharon I, Castelle CJ, Probst AJ, et al. Thousands of microbial genomes shed light on interconnected biogeochemical processes in an aquifer system. Nat Commun. 2016;7:13219. doi: 10.1038/ncomms13219. PubMed PMID: 27774985; PubMed Central PMCID: PMCPMC5079060.

13. Edgar RC. MUSCLE: multiple sequence alignment with high accuracy and high throughput. Nucleic Acids Res. 2004;32(5):1792-7. doi: 10.1093/nar/gkh340. PubMed PMID: 15034147; PubMed Central PMCID: PMC390337.

14. Larsson A. AliView: a fast and lightweight alignment viewer and editor for large datasets. Bioinformatics. 2014;30(22):3276-8. doi: 10.1093/bioinformatics/btu531. PubMed PMID: 25095880; PubMed Central PMCID: PMCPMC4221126.

15. Buchfink B, Xie C, Huson D. Fast and sensitive protein alignment using DIAMOND. Nat Methods. 2015;12(1).

16. Orsi WD, Richards TA, Francis WR. Predicted microbial secretomes and their target substrates in marine sediment. Nat Microbiol. 2018;3(1):32-7. doi: 10.1038/s41564-017-0047-9. PubMed PMID: 29062087.

17. Salter S, Cox M, Turek E, Calus S, Cookson, WO, Moffatt M. Reagent and laboratory contaminantion can critically impact sequence-based microbiome analyses. BMC Biol. 2014;12(82):1-12. doi: 10.1186/s12915-014-0087-z.

18. Blair CC, D'Hondt S, Spivack AJ, Kingsley RH. Radiolytic hydrogen and microbial respiration in subsurface sediments. Astrobiology. 2007;7(6):951-70. doi: 10.1089/ast.2007.0150. PubMed PMID: 18163872.

19. Sauvage J, Flinders A, Spivack AJ, Pockalny R, Dunlea AG, Anderson CH, et al. The contribution of water radiolysis to marine sedimentary life. Abstract EP54B-04, 2019 Fall Meeting, AGU, San Francisco, CA. *2019*.
